# The Role of TLR4 in the Paclitaxel Effects on Neuronal Growth In Vitro

**DOI:** 10.1371/journal.pone.0056886

**Published:** 2013-02-18

**Authors:** Elena E. Ustinova, Galina V. Shurin, Dmitriy W. Gutkin, Michael R. Shurin

**Affiliations:** Department of Pathology, University of Pittsburgh Medical Center, Pittsburgh, Pennsylvania, United States of America; Cincinnati Childrens Hospital Medical Center, United States of America

## Abstract

Paclitaxel (Pac) is an antitumor agent that is widely used for treatment of solid cancers. While being effective as a chemotherapeutic agent, Pac in high doses is neurotoxic, specifically targeting sensory innervations. In view of these toxic effects associated with conventional chemotherapy, decreasing the dose of Pac has been recently suggested as an alternative approach, which might limit neurotoxicity and immunosuppression. However, it remains unclear if low doses of Pac retain its neurotoxic properties or might exhibit unusual effects on neuronal cells. The goal of this study was to analyze the concentration-dependent effect of Pac on isolated and cultured DRG neuronal cells from wild-type and TLR4 knockout mice. Three different morphological parameters were analyzed: the number of neurons which developed neurites, the number of neurites per cell and the total length of neurites per cell. Our data demonstrate that low concentrations of Pac (0.1 nM and 0.5 nM) do not influence the neuronal growth in cultures in both wild type and TLR4 knockout mice. Higher concentrations of Pac (1–100 nM) had a significant effect on DRG neurons from wild type mice, affecting the number of neurons which developed neurites, number of neurites per cell, and the length of neurites. In DRG from TLR4 knockout mice high concentrations of Pac showed a similar effect on the number of neurons which developed neurites and the length of neurites. At the same time, the number of neurites per cell, indicating the process of growth cone initiation, was not affected by high concentrations of Pac. Thus, our data showed that Pac in high concentrations has a significant damaging effect on axonal growth and that this effect is partially mediated through TLR4 pathways. Low doses of Pac are devoid of neuronal toxicity and thus can be safely used in a chemomodulation mode.

## Introduction

Paclitaxel (Pac), a diterpene purified from the bark of the western Yew (*Taxus brevifolia*), is an antitumor agent that is widely used for treatment of breast, lung and other solid cancers [1], [2], [3]. Pac primarily exerts its effect by binding to the β-tubulin subunit in microtubules, preventing depolymerization and increasing their stability and rigidity. This leads to enhanced polymerization, cell-cycle arrest and enhanced apoptosis of proliferating cells [4]. In addition, Pac was shown to be a ligand to TLR4, a member of the Toll-like receptor family, a class of pattern recognition molecules in innate and acquired immune responses [5]. Expression of TLR4 was also detected in several tumors and tumor cell lines and was shown to be involved in the anti-proliferative action of Pac [6]. Recently TLR4 was found to be expressed on central and peripheral neurons [7], [8], [9]; however, the functional impact of TLR4 signaling in neurons remains to be determined. TLR4-deficient mice demonstrated enhanced neural stem/progenitor cell proliferation and neuronal differentiation [10]. In addition, TLR4-deficient mice displayed increases in diffuse amyloid beta-protein and its deposits compared with WT mice [11], suggesting that TLR4 signaling might be involved in Alzheimer's disease progression and could be a new therapeutic target for Alzheimer's disease [12].

While being effective as a chemotherapeutic agent, Pac in high therapeutic doses (MTD, maximum tolerated doses) has several significant side effects. Peripheral neuropathy is the major adverse effect associated with the therapeutic use of Pac and this has been demonstrated to be dependent upon the dose administered, the duration of the infusion, and the schedule of administration. The symptoms of sensory loss and paresthesia can persist even following cessation of treatment with Pac and may significantly impact on quality of life of patients with cancer [13], [14]. The development of neuropathy is dose-dependent and appears to be more prevalent with dose-dense regiments. The symptoms often occur in anatomical structures innervated by the longest nerves in a symmetrical manner predominantly affecting the distal hands and feet [15]. This suggests that Pac may target the cell bodies of sensory neurons. In fact, preferential accumulation of Pac has been reported in the dorsal root ganglia (DRGs) following intravenous administration in rodents [16]. In-vivo studies have suggested that Pac produces neurotoxicity via interruption of microtubule dynamics leading to overstabilization and subsequent axonal transport dysfunction [17]. With prolonged dysfunction, chronic Pac administration may eventually lead to axonal degeneration via multiple mechanisms, including calcium-mediated calpain cascade activation, mitochondrial dysfunction, and long-term effects of axonal transport deficits [14].

Although the use of Pac as a chemotherapeutic agent has become a broadly accepted option in the treatment of patients with different solid tumors, significant toxicities, such as peripheral neuropathy and myelosuppression, limit the effectiveness of Pac-based treatment regimens. MTD-based Pac chemotherapy can efficiently suppress the generation and function of the immune cells leading to increased susceptibility to infectious diseases and collapse of the anti-tumor immunity [18], [19], [20]. In view of these undesirable toxic effects associated with conventional chemotherapy, decreasing the dose of Pac has been recently suggested as an alternative approach, which might limit neurotoxicity and immunosuppression [21]. For instance, it has been recently reported that Pac in low and ultra low doses directly stimulates activity of immune effector cells, increases their ability to recognize cancerous cells [22], [23], and up-regulates the efficacy of anticancer immunotherapy in vivo [24]. Interestingly, Pac in ultra low doses may also decrease the number and activity of immunosuppressive immune regulatory cells both in vivo and in vitro [25], [26]. This new phenomenon, i.e., immunostimulatory properties of certain chemotherapeutic agents in ultra low doses, has been called ‘chemomodulation’ [21], [27], [28] and suggests that certain chemotherapeutic drugs, including Pac, may exhibit unexpected noncytotoxic/noncytostatic activities when used in ultra low doses. However, it remains unclear if low and ultra low doses of Pac retain its neurotoxic properties or might exhibit unusual effects on neuronal cells.

The goal of this study was to analyze the dose-dependent effects of Pac on neuronal cells in a mouse DRG model in vitro and its dependability on TLR4 signaling. We have demonstrated a marked negative effect of high concentrations of Pac on neurite growth, which was partially mediated by the TLR4 signaling. In contrast, low concentrations of the drug did not show any neuronal impairment.

## Materials and Methods

### Animals

Six- to eight-week-old male C57BL/6 mice (Taconic, Germantown, NY) and C57BL/10ScNJ mice with a deletion of the *Tlr4* gene (Jackson Lab, Bar Harbor, Maine) were housed in a pathogen-free facility under controlled temperature, humidity, and 12-h light/dark cycle with a commercial rodent diet and water available *ad libitum*. Experimental protocols were approved by University of Pittsburgh Institutional Animal Care and Use Committees.

### DRG cultures

DRG dissection and separation was performed according to Malin et al. (2007) with small modifications [29]. Briefly, the mice were sacrificed by carbon dioxide inhalation and DRG ganglions were dissected immediately from the spinal column and collected in Ca^++^/Mg^++^- free HBSS (Invitrogen, Grand Island, NY). Ganglia were incubated at 37°C with 60 units of papain for 10 min followed by 20 min incubation with the mixture of 4 mg/ml collagenase-2 and 4.5 mg/ml neutral peptidase (all from Worthington Biochemical Corporation, Lakewood, NJ). Enzymatically dissociated ganglia were washed in F-15 medium (Invitrogen, Grand Island, NY) and gently triturated by a series of pipetting with decreasing tip diameter. Dissociated neurons were resuspended in F-15 medium containing 10% heat-inactivated FCS and 1% penicillin/streptomycin (10,000 U/ml). Cell suspension (150 µl) was distributed on rounded glass coverslips pre-coated with poly-d-lysine (10 µg/ml)/laminin (200 µg/ml) (all from Sigma-Aldrich, St. Louis, MO) and placed in 12-well culture plates. Two hours later all wells were filled with additional 850 µl of complete F-15 medium. Pac (Mayne Pharma, Salisbury South, Australia) was added to neuronal cultures at final concentrations of 0.1 nM–100 nM.

Neurons were cultured for 48 hours at 37°C in 5% CO_2_. At 24 hours, 75% of culture medium was replaced with the fresh medium containing the same concentrations of Pac. Forty-eight hours after plating, coverslips with cultured neurons were fixed in 2% paraformaldehyde, stained with brilliant blue stain (Sigma) and mounted on the glass slides.

In separate series of experiments we evaluated the effects of TLR4 inhibitor [30] and TLR4 agonist, lipopolysaccharide (LPS) on the neurons from wild type animals, treated and untreated with PAC. LPS-RS Ultrapure (5 µg/ml) (InvivoGen, San Diego, CA), LPS (0.5 µg/ml) (Sigma-Aldrich, St Louis, MO) and PAC (100 nM) were added to the neuron media 2 hours after plating, as described above.

### Cytotoxicity Assay

Effect of Pac on the viability of neuronal cells was tested using aCella-Tox kit (Cell Technology, Mountain View, CA). Briefly, the cells were plated at 2000 cells per well and treated with PAC as described above. After 48 hours 100 µl of supernatant was collected from the well and transferred to the white opaque 96 well plate in triplicates. Then, 10 µl of lytic agent was added to the cells for 15 min and another 100 µl sample (positive control–total lysis) was also transferred to the 96 well plate. 100 µl of Enzyme Assay Reagent containing Gyceraldehyde 3-Phosphate was then added to all wells followed by 50 µl of the detection reagent. The plate was immediately read using luminometer (Synergy HT, Biotek). Cytotoxicity was calculated as:



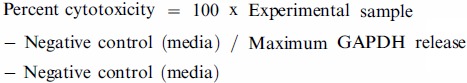



### Analysis of neurite growth

Slides were prepared in triplicates for all tested concentrations of Pac. Twelve randomly selected fields on each slide were photographed at 400X magnification. Images were analyzed with the ImageJ software package (Rasband WS, ImageJ, NIH, http://imagej.nih.gov/ij). Neurites were traced and their lengths were measured with NeuronJ plugin [31]. Cell process exceeding 2 body lengths was considered a neurite. The percentage of DRG neurons with neurites, total length of neurites, and the number of neurites per cell were calculated for each field by two investigators, blinded to the neuron treatment. No less than 40 cells were analyzed per slide, with the average of 1030 cells per slide.

### Statistical analysis

Statistical evaluation of the difference in the number of neurons developing neurites, neurite lengths and the number of neurites per cell was performed using the GraphPad Prism statistical software (GraphPad Software, San Diego, CA). For all analyses, the level of significance was set at probability of p<0.05. ANOVA was used for comparison of multiple groups. For single comparison of two groups, the Student *t*-test was used after evaluation of normality. All experiments were repeated at least 3–4 times. Data are presented as the means ± SEM.

## Results

### Effect of paclitaxel on neuron viability

Pac in the concentrations 0.1–100 nM had no effect on the neuron viability. The percent of neuronal death after 48 hours in culture was 12±3% in the absence of Pac and 16±4% at the highest concentration of paclitaxel of 100 nM.

### Concentration-dependent effects of paclitaxel on cultured neurons

Three different morphological parameters were analyzed in each slide: the number of neurons which developed neurites, the number of neurites per cell and the total length of neurites per cell. The first two of these parameters reflect the process of growth cone initiation and the third one reveals the process of neurite extension.

**Figure 1:**
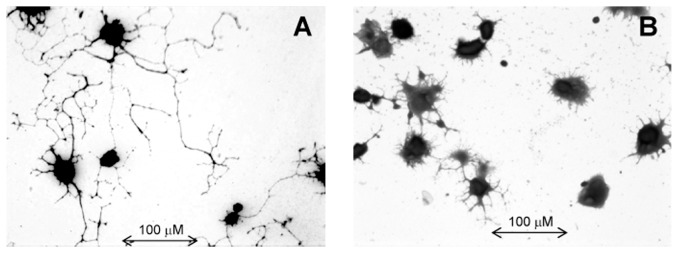
Cell morphology of neurons in DRG cultures (high power view). **A**. In control cultures, neurons develop numerous well defined neurites with multiple extensive branching after 48 h of incubation. **B**. Neurons incubated with high concentrations of Pac (100 nM) develop multiple short processes, but no well-formed neurites. Representative pictures are shown from >5 independent experiments with similar results.

As expected, immediately after harvesting and plating the neurons appear as round cells with no neurites. After 24 hours in Pac-free cultures, the process of neurite development was initiated. At 48 hours, the vast majority of cultured neurons (70.8±3.5%) have numerous well defined neurites with an extensive branching (Figure 1). The average length of neurites per cell was 1095±115 µm.

**Figure 2:**
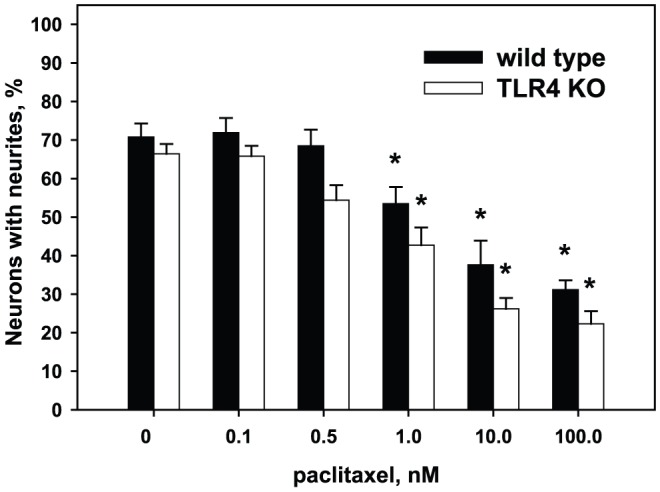
Effects of paclitaxel on the number of neurons with neurites in control and treated DRG cultures. DRG cultures were prepared from wild type and TLR4 knockout animals as described in Materials and Methods. Pac was added to cultures at concentrations 0.1 nM–100 nM. As shown, Pac reduces the number of regenerating neurons in a concentration-dependent manner, both in DRG cultures obtained from control wild type (solid bars) and TLR4 knockout mice (open bars). *, p<0.05 versus 0 M of Pac in the same group (N = 5, one-way ANOVA).

**Figure 3:**
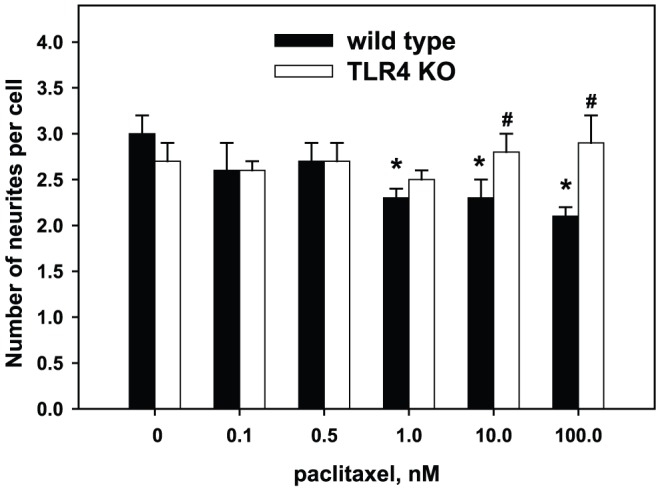
Effects of paclitaxel on the number of neurites per cell in control and treated DRG cultures. DRG cultures were prepared from wild type and TLR4 knockout mice as described in Materials and Methods. Pac was added to cultures at concentrations 0.1 nM–100 nM. Pac inhibits neurite development in a concentration-dependent manner in wild type mice (solid bars), but not in TLR4 knockout mice (open bars). *, p<0.05 versus 0 M of Pac in the same group; #, p<0.05 vs. wild type at the same concentration of Pac (N = 5, one-way ANOVA).

**Figure 4:**
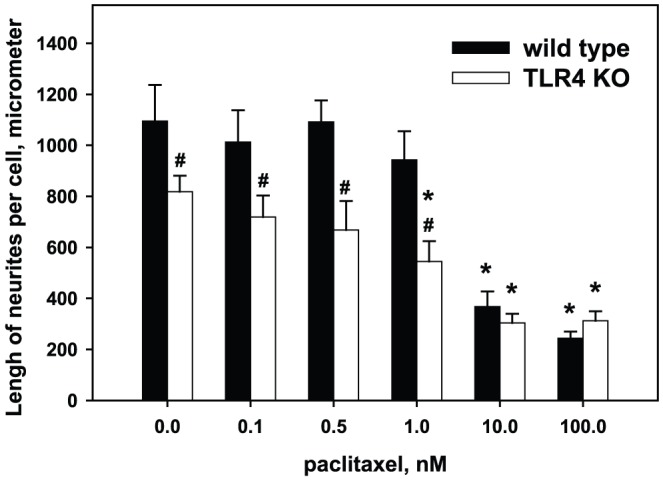
Effects of paclitaxel on the total length of neurites per cell in control and treated DRG cultures. DRG cultures were prepared from wild type and TLR4 knockout animals as described in Materials and Methods. Pac was added to cultures at concentrations 0.1 nM–100 nM. As shown, Pac decreases the length of neurites in a concentration-dependent manner both in control (solid bars) and TLR4 knockout mice (open bars). *, p<0.05 versus 0 M of Pac in the same group; #, p<0.05 versus wild type at the same concentration of Pac. (N = 5, one-way ANOVA).

In DRG neurons isolated from wild type animals, Pac in low concentrations (0.1 nM and 0.5 nM) affected neither the number of regenerating neurons nor the total number and length of neurites per cell (p>0.05). In contrast, high concentrations of Pac showed a significant concentration-dependent inhibition of neurite growth by decreasing the number of regenerating neurons, the number of neurites per cell, and the total length of neurites. Morphologically, neurons develop numerous short processes, only few of which continue to grow and present as well-formed neurites. Specifically, at the concentration of 1 nM only 53.2±6.1% of neurons developed neurites (p<0.05 vs control), and at the high concentrations of 10 nM and 100 nM only one third of neurons had neurites (28.5±4.3%, p<0.05 vs control) (Figure 2). The number of neurites per cell was lower at concentrations of 1 nM–100 nM (Figure 3). For instance, in the absence of Pac neurons developed 3.0±0.2 neurites per cell and at the concentration of 1 nM and 100 nM of Pac only 2.3±0.1 and 2.1±0.1 neurites per cell, respectively (p<0.01). The average length of neurites per cell was significantly decreased at the high concentrations of 10 nM and 100 nM, but was not affected at the concentration of 1 nM (Figure 4). For example, at the concentration of 1 nM the lengths of neurites per cell was 943±112 µm vs.1096±142 µm in the absence of Pac (p = 0.1). At the concentration of 10 nM and 100 nM the lengths of neurites per cell was 368±59 µm and 244±23 µm, respectively (p<0.001).

Thus, these data demonstrate that in DRG neurons from wild type animals Pac does not affect neuronal growth at low concentrations (0.1–0.5 nM), but significantly decreases all parameters of neuronal growth at the high concentrations of 10–100 nM.

### Effects of paclitaxel on neurons obtained from TLR4 knockout mice

As shown on Figure 2, in the absence of Pac neurons from TLR4 knockout mice had approximately the same proportion of cells that develop neurites as the neurons from wild type mice (66.4±2.3% vs. 70.8±3.5%, p = 0.14). The number of neurites per cell in Pac-free cultures also was not different from wild type mice (2.7±0.2 vs 3.0±0.2, p = 0.14) (Figure 3). However, the average length of neurites in Pac-free cultures from TLR4 knockout mice was significantly lower than in the cultures from wild type mice (818±63 µm vs.1096±141 µm, p = 0.03) (Figure 4). Low concentrations of Pac (0.1 nM and 0.5 nM) did not influence the proportion of cells with neurites and the average length of neurites in cultures obtained from TLR4-deficient mice. High concentrations of Pac (10 nM and 100 nM) significantly decreased the proportion of cells developing neurites (26.2±2.8% and 22.3±3.5%, respectively; p<0.05 vs 0 nM Pac) and the average length of neurites (304±36 µm and 312±36 m, respectively; p<0.05 vs 0 nM Pac) in the same manner as in the cultures from wild type animals.

Interestingly, in contrast to wild type animals, Pac did not influence the number of neurites per cell in cultures prepared from TLR4-deficient mice even at the high concentrations. The number of neurites per cell at the highest concentration of Pac of 100 nM was 2.9±0.4 vs 2.7±0.1 in Pac-free medium. At the concentrations of 10 nM and 100 nM neurons from the TLR4 knockout mice had significantly more neurites per cell than neurons from wild-type animals at the same concentrations of Pac: 2.8±0.2 vs 2.3±0.2 (p<0.05) at 10 nM and 2.9±0.4 vs 2.1±0.1 (p<0.05) at 100 nM.

### Effect of LPS-RS on paclitaxel-treated neurons

**Figure 5:**
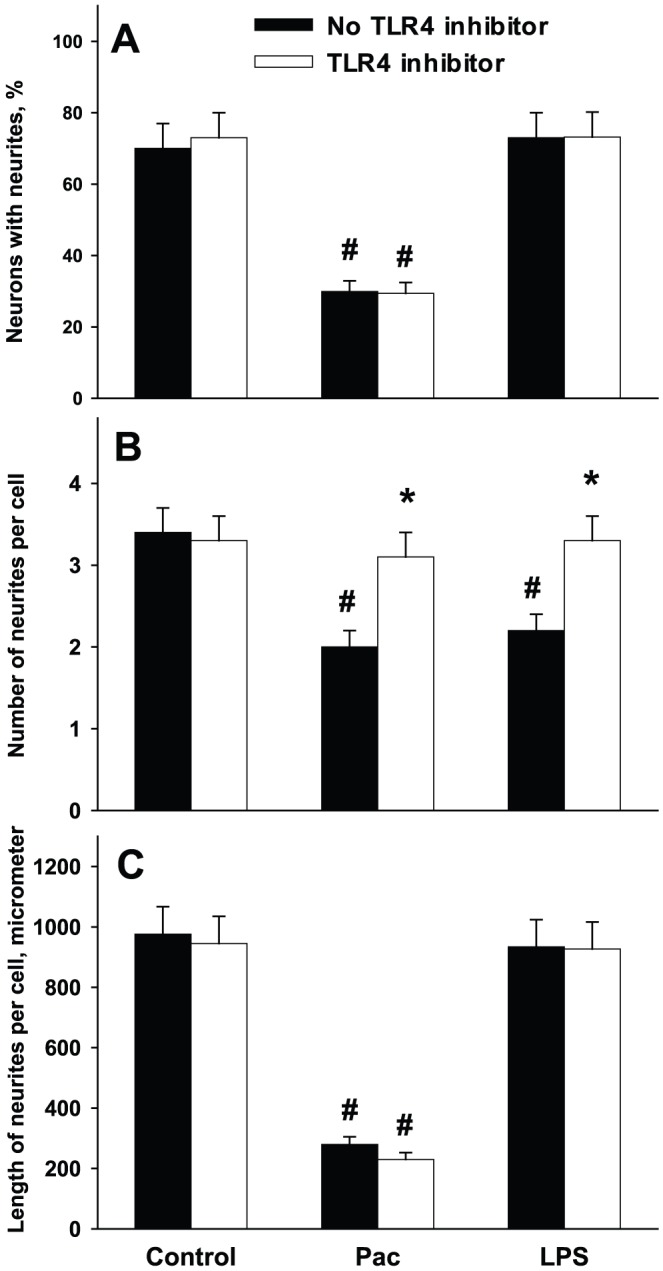
Effect of TLR4 inhibitor LPS-RS on the morphological parameters of control, paclitaxel-treated and LPS-treated neurons. DRG cultures were prepared from wild type animals as described in Materials and Methods. Pac was added to cultures at 100 nM; LPS at concentration of 0.5 µg/ml and LPS-RS at concentration of 5 µg/ml. A, percent of neurons with neurites; B, number of neurites per cell; C, total length of neurites per cell. *, p<0.05 versus no LPS-RS; #, p<0.05 versus respective control group (N = 5, one-way ANOVA).

To further evaluate the role of TLR4 signaling in Pac-induced inhibition of neurite growth we attempted to block its action by using TLR4 inhibitor LPS-RS. As shown on Figure 5, TLR4 inhibitor does not influence the number of neurons with neurites, or the length of neurites after Pac treatment. However, the number of neurites per cell is significantly higher in LPS-RS group after Pac treatment. These data show the protective effect of LPS-RS on the process of growth cone initiation in Pac-treated neurons. To confirm that LPS-RS exerts its action through TLR4 inhibition, we evaluated the effect of TLR4 agonist (LPS) on cultured neurons. As shown on Figure 5, LPS decreases the number of neurites per cell, but does not influence other morphologic parameters. The effect of LPS is completely abolished by LPS-RS.

In summary, our results revealed that Pac in low concentrations did not affect the neuronal growth in DRG cultures prepared from both wild type and TLR4 knockout mice. The effect of Pac at high concentrations on the length of neurites did not depend on the presence of TLR4, whereas the effect on the number of developing cell processes was different in wild type and TLR4-deficient mice suggesting the possible role of TRL4 signaling in neurite development.

## Discussion

Paclitaxel exerts its cellular effects through several different mechanisms. The first one, which has been shown for numerous cell types, consists of Pac binding to beta-tubulin [32]. This leads to over-stabilization of microtubules and disruption of vital functions such as cell division, maintenance of cell shape and intracellular transport [33]. Another mechanism is an interaction of Pac with TLR4 receptor, which produces different cellular effect, depending on the type of cells involved [34], [35]. Activation of the TLR4 signaling by Pac has been demonstrated in murine macrophages, transfected cell lines, and mouse tumor cell lines [35], [36], although the results revealing involvement of TLR4 on the Pac effects on myeloid cells are controversial [19] and might be concentration-dependent [28].

Paclitaxel is widely used in oncology clinic, but the therapeutic concentrations are neurotoxic, specifically targeting sensory innervations. Pac-induced neuropathy is characterized by prominent paresthesias and dysesthesias with abnormal functions of both large and small sensory fibers [13], [37]. Sensory nerve dysfunction is typically more common than motor involvement, perhaps reflecting the vulnerability of sensory neuronal cell bodies in the DRG to toxic damage. The prominent vascularization of the DRG and permeability of the blood-nerve barrier may support the accumulation of toxic compounds in sensory neurons [14]. One can assume that Pac causes neuronal damage through its two known pathways, but our understanding of the mechanisms behind the neurotoxic effects of Pac is incomplete. Adult peripheral neurons do not proliferate, and thus, the anti-mitotic activity of Pac would not be a damaging factor. However, the maintenance of axonal integrity and function requires constant remodeling that involves regulation of axonal microtubular structures [38]. In microtubules, tubulins are polymerized at one end and are depolymerized at the other end constantly. Dissociated tubulins and formed microtubules exist in equilibrium. This equilibrium is necessary for axonal transport [13]. Systemic Pac exposure in vitro induces marked microtubule aggregation in large myelinated axons and DRG neurons [39], [40]. These data indicate that the first mechanism of Pac activity, i.e., tubulin binding, is an important factor of its neurotoxicity. This mechanism is shared by other taxanes (docetaxel) and other types of microtubule-stabilizing agents, like epothilones, peloruside, etc [17], [41]. On the other hand, the role of TLR4-mediated effects of Pac on neurons has not yet been evaluated.

In this study we have demonstrated a direct effect of Pac on adult somatic neurons in murine DRG cultures. DRG culture is an accepted model for evaluation of potential toxicity of different chemotherapeutic drugs [42], [43], [44]. Our results showed that high concentrations of Pac caused a significant damage of DRG neurons taken from wild type mice. All of the morphologic parameters that have been evaluated were markedly affected, including the number of viable neurons, number of neurites per cell, and the length of neurites. These results are in agreement with earlier studies performed on murine, rat, and chicken neuronal cultures [42], [43], [44].

The length of neurites of DRG neurons from TLR4 knockout mice was inhibited to a similar extent as for wild type neurons; however, the number of neurites per cell, indicating the process of growth cone initiation, was not affected by either low or high concentrations of Pac. Thus, our data prove for the first time that Pac in high concentrations exerts its noxious effect on neurons through two different mechanisms: tubulin-binding and TLR4 signaling. Its tubulin targeting activity is likely to be responsible for a prevention of neurite outgrowth and elongation. It was demonstrated previously that the neurite growth depends on polymerization and depolymerization of microtubules that is directly affected by Pac [13]. On the other hand, Pac effect on a process of growth cone initiation depends on TLR4 signaling, since cells from TLR4 knockout mice were protected against it. TLR4-stimulating activity can be unique to Pac, since other microtubule-stabilizing compounds, such as peloruside, docetaxel, and epothilone B do not show this effect in animal models [45]. Pac effect on growth cone initiation was also abolished in wild-type animals with the use of LPS-RS. This substance exerts its effect through binding to MD-2 co-receptor, thus blocking TLR4-dependent signaling [30]. The role of TLR receptors in neuronal development is not clear, however, experimental data demonstrate that signaling through TLR might causes growth cone collapse in central and peripheral neurons in mice [46].

We have also shown that Pac in low and ultra-low concentrations does not affect neuronal morphology and neurite growth. Although these results are expected, they have a direct practical significance, since low-dose chemotherapy finds its way into clinical practice. A new therapeutic approach, called chemomodulation, is based on an ability of certain chemotherapeutic agents, including Pac, to enhance tumor immunogenicity without inducing tumor cell apoptosis, and thus enhance the efficacy of immunotherapy. Recently, we and others have shown that Pac in low noncytotoxic doses exhibits multiple immunomodulating activities, including tumor cell recognition by tumor-specific cytotoxic T cells, enhanced tumor antigen presentation by dendritic cells, up-regulation of maturation of dendritic cells and IL-12 production, suppression of accumulation and function of protumorigenic myeloid-derived suppressor cells and regulatory dendritic cells, and increase of efficacy of anti-tumor vaccination protocols [22], [23], [26], [27], [28], [47]. The fact that Pac in low doses does not exhibit the neurotoxic activities makes new chemomodulation approaches even more clinically attractive and feasible.

In summary, our data on murine DRG cultures showed that tubulin-stabilizing agent paclitaxel in high concentrations has a significant damaging effect on axonal growth and that this effect is partially mediated through TLR4 pathways. Low concentrations of Pac are devoid of neuronal toxicity and thus can be safely used in a chemomodulation mode. Future studies in the field should reveal dose-dependent neurotropic effects of other microtubule-binding agents and confirm their effects on neurons in the in-vivo setting.
